# Modulation of spontaneous proliferation of T-lymphocytes from HTLV-1- infected individuals by quinoline compounds

**DOI:** 10.1186/1742-4690-11-S1-P121

**Published:** 2014-01-07

**Authors:** Lorena A Pinto, Marcos A Vannier-Santos, Alain Fournet, Bernardo Galvão-Castro, Maria FR Grassi

**Affiliations:** 1 Gonçalo Moniz Research Center - CPqGM/FIOCRUZ, Salvador, Ba, Brazil; 2 Faculté de Pharmacie, rue J. B. Clément, Châtenay-Malabry Cedex, France; 3 Bahiana School of Medicine and Public Health, Brazil

## 

Spontaneous proliferation, a hallmark of Human-T Lymphocyte Virus Type 1 (HTLV-1) infection, is particularly higher in HAM/TSP patients compared to asymptomatic carriers. However the role of the proliferation in the pathogenesis of HAM/TSP is still unknown. The identification of drugs that modulate the spontaneous proliferation may be important for the treatment of HAM/TSP disease. We have evaluated the effect of three different quinoline compounds (A, B and C) on the modulation of spontaneous proliferation of peripheral blood mononuclear cells from HTLV-1 patients with HAM/TSP. The cells from 8 HAM/TSP patients were cultivated in the presence of serial concentrations of quinolone compounds. Cell proliferation was assessed by 3[H]thymidine incorporation. Cell viability was measured by optical density in the presence of MTT. The ultrastructure analysis was done using a transmission electron microscope. Quinoline compounds were not toxic at the concentrations evaluated. The IC50 was 18.5 µM for compound A, 30.5 µM for compound B and 3.4 µM for compound C. The three compounds inhibited more than 90% of spontaneous proliferation. Cultured cells in the presence of quinolone compounds showed vacuoles presented with myelin-like membranes, probably autophagic vacuole-like compartments, observed by electronic microscopy. In conclusion, the quinoline compounds showed no toxicity and were able to inhibit the spontaneous proliferation of T cells from HTLV-1-infected individuals. New assessments are being applied to understand how the quinoline compounds act on cells by decreasing cell proliferation.

